# An emerging clock mechanism in a hydrozoan jellyfish

**DOI:** 10.1371/journal.pbio.3003558

**Published:** 2026-01-07

**Authors:** Ezio Rosato

**Affiliations:** Division of Genetics and Genome Biology, School of Biological and Biomedical Sciences, College of Life Sciences, University of Leicester, Leicester, United Kingdom

## Abstract

In hydrozoan jellyfish, the timing of gamete release is often coordinated by light. This primer discusses findings from a recent PLOS Biology article that elucidates a novel, endogenous clock-based mechanism that governs egg release in a new species of jellyfish.

## Teaser

The timed release of gametes is an important feature of marine organisms, and in hydrozoan jellyfish is usually controlled by light. A recent study in *PLOS Biology* reveals an emerging endogenous clock controlling rhythmic egg release in a novel hydrozoan species.

For many marine species that fertilize externally, eggs, and sperm must find each other in the open ocean, while avoiding inter-species mixing. Light-dark cycles provide an obvious synchronizer, being a common cue which all individuals can align to [1]. Moreover, synchronization allows species to occupy distinct temporal niches within the same area (like reserving a table at the restaurant), reducing the risk of inter-crossing.

A timing mechanism that relies on environmental cues resembles an hourglass. The stimulus sets the time (turns the hourglass), and at the end of the count-down a new stimulus is required to initiate another cycle. This type of mechanism can explain the rhythmic ovulation of many hydrozoan jellyfish. For instance, in *Clytia hemisphaerica*, a light stimulus triggers the secretion of a maturation-inducing hormone (MIH) from the gonads, which initiates meiotic division in the oocytes and the release of eggs. Predictably, the MIH-secreting cells express opsins (light-sensitive proteins) [2].

In this issue of *PLOS Biology*, Kitsui and colleagues describe a novel species, *Clytia* sp. IZ-D, a close relative of *C. hemisphaerica.* In this species, eggs are released not as a direct reaction to light but in response to an endogenous clock [3]. It is the first time such a phenomenon is described in hydrozoans.

Firstly, the authors noticed that under a 12-hours light: 12-hours dark (LD 12:12) cycle (at 21 °C) mature oocytes (that had completed meiosis) were released two hours after the light-to-dark transition. Altering the onset of the dark period did not change the timing of ovulation, whereas advancing the onset of the light period did. This suggested that the trigger for ovulation was the lights-on switch, not the onset of darkness. Indeed, under different photoperiods spawning occurred always 14 hours after lights-on, even when illumination lasted just 1 min. Such observations are difficult to reconcile with an hourglass model. Additionally, the authors noticed that in each female spawning lasted for up to 60 min in *Clytia* sp. IZ-D, whereas *C. hemisphaerica* completed ovulation within 10 min. Thus, in the former species, the timing of maturation probably depends on individual oocytes, at least in part.

Rhythmicity in a living system may reveal a biological clock, endogenous and self-sustained. Unlike an hourglass, a clock does not require a rhythmic environmental signal to initiate a cycle. Instead, the signal functions as an entrainment cue, forcing the clock to align its phase to it. Additionally, the period of a clock does not depend on temperature. While an hourglass relies on a single counting-down process, the architecture of a clock is built upon multiple interconnected mechanisms, whose interactions compensate the effect of temperature.

**Fig 1:**
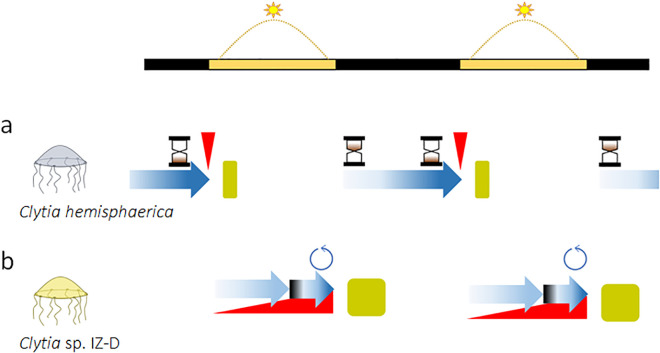
Rhythmic ovulation in *Clytia species*. **(a)** In *Clytia hemisphaerica*, the oocytes develop (blue arrow) becoming competent to respond to the maturation-inducing hormone (MIH), which triggers meiosis. MIH (red triangle) is released by neurosecretory cells in the gonads when they become exposed to light. After completion of meiosis (about 2 hours), the mature eggs are released. Ovulation (green square) lasts about 10 min. Since light is required to start a cycle, whose duration is regulated by the developmental programme, in *C. hemisphaerica* rhythmic ovulation is analogous to an hourglass. **(b)** In *Clytia* sp.IZ-D light is required for synchronous development of the oocytes (blue arrow). However, the final maturation process, which makes them responsive to MIH, is gated by an unknown mechanism that likely operates individually in each oocyte (black-and-blue arrow). Light also drives the accumulation and progressive release of MIH. Meiosis begins when the oocytes reach the right maturation stage and MIH the right concentration. After about 2 hours ovulation begins, lasting about 60 min (green box). Together, the timing mechanisms controlling oocyte development and MIH release constitute an incipient clock. Two light-dark cycles are shown for reference.

To demonstrate the presence of a clock in *Clytia* sp. IZ-D, the authors tested whether the ovulation cycle was endogenous, self-sustained, and temperature compensated. Using constant light, constant darkness, and different temperatures, they demonstrated that rhythmic ovulation was endogenously driven but light-dependent (as it persisted under constant light but not constant darkness), was entrainable by a light–dark cycle, but was not temperature compensated. Thus, in *Clytia* sp. IZ-D, ovulation is no longer controlled by an hourglass (like in *C. hemisphaerica*) but neither by a fully-fledged circadian clock. The authors then designed a series of clever physiological experiments to investigate how such a clock could emerge from the hourglass mechanism. In *C. hemisphaerica*, light triggers the release of MIH from the opsin-positive neurosecretory cells of the gonads, at a time when the oocytes are ready to respond to it engaging in meiosis. Since the rhythmic light stimulus becomes effective only after the oocytes have reached full development (the count-down process), this explains the hourglass mechanism. In *Clytia* sp. IZ-D, the oocytes require light to develop synchronously in the gonads up to a point (stage III) when they prepare for meiosis, which is triggered by MIH. However, unlike *C. hemisphaerica*, this event is gated, meaning there is a block in the progression of development stopping the oocytes being responsive to MIH. The duration of the gate depends on an (unknown) endogenous process modulated by light and temperature, and likely executed individually in each oocyte. Additionally, light does not trigger the immediate release of MIH. Instead, it initiates a program (unknown) that may result in the progressive accumulation and release of the hormone. Thus, the introduction of a gate separating two phases of oocyte development and the addition of a delay in the production/release of MIH have moulded the hourglass into a *quasi*-clock (Fig 1).

In its essence, a circadian clock is composed of an effector (producing the output) and a modulatory process. What bends two linear activities into a cycle is the timing of their interaction. There must be a period when the two processes cannot interact (delay) and that interval must be determined by independent (but cooperative) and offsetting activities [4]. The best-known clocks are based on transcription translation feedback loops (TTFLs), but it is the organisation, not the actual elements, what makes the clock.

In *Clytia* sp. IZ-D, light is required to synchronise the development of the oocytes in the gonads, to time their final individual maturation, and to initiate MIH production and release. Both light and temperature speed up these processes. Thus, the modulatory elements do not compensate each other, explaining why such a clock “fails” the temperature-compensation test.

In summary, the article by Kitsui and colleagues describes a phenomenon that goes well beyond the immediate interest of hydrozoan biology. Many details remain to be clarified, not least the identity of the molecular controls. Cnidaria (which includes Hydrozoa) possess the ancestral constituents of the TTFL, such as Clock, Bmal1/Cycle and Cryptochrome [5,6]. Conversely, they do not have Period and (true) Timeless, which are bilaterian innovations that likely conferred robustness to the clock. However, hydrozoans stand apart, since, independently, they have lost Clock and Bmal1/Cycle [7]. Future work should address whether other molecular components compensate for the loss of these major elements of the TTFL, or an altogether novel clock mechanism has evolved in this fascinating group of animals.

## Abbreviations:

MIH: maturation-inducing hormone
TTFL: transcription translation feedback loop
